# Multicentric Carpo-Tarsal Osteolysis Syndrome (MCTO) and “Function Profile”: a rehabilitative approach

**DOI:** 10.1186/s13023-023-02976-z

**Published:** 2023-12-20

**Authors:** Anna Bruna Ronchetti, Marina Usai, Valentina Savino, Marco Scaglione, Chiara Maria Tacchino, Marta Bertamino, Paolo Moretti, Maja Di Rocco

**Affiliations:** 1 Physical Medicine and Rehabilitation Unit, IRCCS Istituto Giannina Gaslini, Genoa, Italy; 2 Department of Neuroscience, Rehabilitation, Ophthalmology, Genetics and Maternal-Child Sciences, University of Genoa, Genoa, Italy; 3 Rare Diseases Unit, IRCCS Istituto Giannina Gaslini, Genoa, Italy

**Keywords:** Multicentric carpo-tarsal osteolysis, ICF-CY, Clinical outcome measures, Physiotherapy, Occupational therapy, Rehabilitation

## Abstract

**Background:**

Multicentric Carpo-Tarsal Osteolysis Syndrome (MCTO) is an autosomal dominant disease with increased bone reabsorption in the carpus and tarsus and the elbows, knees and spine. The disease is extremely heterogeneous and secondary and tertiary injuries vary widely and can lead to progressive disability and severe functional limitations. In addition to the available and upcoming drug therapies, physical medicine and rehabilitation are important treatment options. Currently, the indication and plan are overlooked, nonspecific and reported only for one patient.

**Methods:**

We describe a case series of MCTO patients diagnosed and followed by a centre to identify functional deficit as a potential clinical marker of disease progression for future etiological therapies. In addition, we define a symptomatic treatment approach and specific clinical management, including a patient-centred rehabilitation approach. Functional assessments are performed independently by a multidisciplinary group to establish the functional abilities of patients and the relationship between residual motor skills and their degree of autonomy and participation. We suggest a way to identify a rehabilitation plan based on a specific disease using the International Classification of Functioning, Disability and Health Children and Youth (ICF-CY).

**Results:**

To define a reliable and reproducible “Function Profile”, through age and over time, we used to value the disease status according to the ICF-CY domains. It could be used to determine the complexity of the illness, its overall impact on the complexity of the person and the burden on the caregiver, and an eventual short- and long-term rehabilitation plan for MCTO and other ultra-rare diseases.

**Conclusion:**

Based on the MCTO experience, we suggest a way to determine a rehabilitation plan based on a specific disease and patient needs, keeping in mind that often the final point is not recovering the full function but improving or maintaining the starting point. In all cases, each patient at the time of diagnosis requires a functional assessment that must be repeated over time to adjust the course of rehabilitation. The evaluations revealed the importance of early rehabilitation management in enhancing independence, participation and control of stress deconditioning, shrinking of muscle tendons and loss of movement to immobility.

## Introduction

In the last years, genetic advances led to a better knowledge of the biological basis of rare bone diseases and a new therapeutic perspective. Besides available or future drug therapies, physical medicine and rehabilitation are important therapeutic options. Indications of rehabilitation are available only for less rare skeletal diseases like achondroplasia or osteogenesis imperfect [1], but for other rare genetic bone diseases rehabilitation plan is neglected, not specific and reported only for a single patient.

Starting from the experience in an ultrarare disease, Multicentric Carpo-Tarsal Osteolysis Syndrome (MCTO), we propose a way to identify a rehabilitation plan based on specific disease (biological disease mechanism and natural history of the disease) and patient individual needs, aware that often the endpoint is not the recovery of full function but improvement or maintenance of starting point. The statement “specific” rehabilitation is about the patient’s rehabilitation plan, not the disease. The progression of the disability cannot be predicted due to the clinical heterogeneity of MCTO, similar to other genetic disorders.

Therefore, the rehabilitation plan must be specific to the patient, patient-centered, and modified as the disease progresses.

The rehabilitation plan aims to achieve and maintain as much autonomy as possible in meaningful life contexts for patients. The rehabilitation aims are to achieve and maintain as much autonomy as possible in meaningful life contexts for patients with the highest possible quality of life and improve participation in the activity of daily living.

MCTO with or without nephropathy is an autosomal dominant disease due to V-maf musculoaponeurotic fibrosarcoma oncogene ortholog B (*MAFB*) encoding the basic leucine zipper transcription factor MafB, which regulates the receptor activator of nuclear factor-kB ligand (RANKL); the consequence of genetic defect is an increase of bone reabsorption. Usually, osteolysis starts in pediatric age from carpal and tarsal bones with further reabsorption of other hand and feet bones as well as limb bones; severely affected patients develop deformity of interphalangeal, metatarsophalangeal, metacarpophalangeal, wrists elbows and knees joints [2]. Older patients develop defects of mineralization.

The disease is heterogeneous in age of appearance and progression of the same. The secondary and tertiary injuries that can worsen it are varied and can result in progressive disability and severe functional limitations. The disease’s onset in early childhood and being a “moving target” during personal growth and development may make this even more complicated.

Therefore, for every patient is essential an accurate, complete and repeated clinical-functional assessment over time and a multidisciplinary approach to care is essential. In our experience, patients effectively present pain during the process of osteolysis at the onset of the disease [3, 4]. Patients do not complain of pain, but they do have functional limitations once the flare-up phase is over. Clinical assessment to identify a rehabilitation plan cannot be performed during active osteolysis, when there is pain, but only after flare-up episodes to monitor the possible and often unpredictable evolution of this rare disease and to modulate the necessary rehabilitation intervention [5, 6].

We describe a case series of MCTO patients diagnosed and followed by one centre, with the purpose of focusing on tools to capture functional impairment as a possible clinical marker of disease progression in view of future etiological therapies; furthermore, we delineate an approach to symptomatic therapies and specific clinical management including rehabilitation approach (physiotherapy, hydrotherapy and occupational therapy patient-centred).

Functional assessments are carried out by the physiatrist and occupational therapist, independently, in order to establish the patients’ functional abilities and the correlation between residual motor skills and their level of autonomy and participation.

## Method

Among the various possible evaluations, we chose the ones that we felt best enabled us to monitor disability, which was closely linked to the extremely heterogeneous clinical expression of this disease, according to the age of the patients at the time of observation. We thus followed 9 patients between 2 and 46 years old, all characterized by a genetic variant of MAFB. In this paper, we collected all patients from whom we obtained all the necessary data for a complete functional evaluation.

With no specific scale for the assessment and follow-up of this rare disease, we used other assessments to determine the level of impairment and restriction of children’s activity in everyday life. We concentrated on the evaluations of the International Classification of Functioning, Disability and Health: Children and Youth (ICF-CY), including the area of Body function and Structure, as well as the field of Activities and Participation [7].

### ICF domain: body function and structure

The collected data were organized around the following ICF domains during the single monitoring visit.

(a) *Active and Passive Range of Motion (a-p ROM)*—This is a functional clinical balance for all major grade-rated joints (spine, left and right shoulders, elbows, wrists, hands, hips, knees, ankles and feet). Evaluating the ROM and its difference between active and passive excursion not only provides information on the degree of motion present, but also allows assessment of joint condition in general, pain tolerance and, in part, functional capacity. ROM LAG is not a specific pathological condition, but in MCTO, and in other diseases, could be a consequence of joint and tendon damage, adhesions or significant muscle weakness.

(b) *Manual Muscle Testing (MMT)*—It is a functional clinical balance for all major muscle groups. This involves manual evaluation of individual and group muscle strength (upper and lower limbs, hands and spine). In patients with MCTO, weakness or asymmetry in muscle groups are usually secondary consequences of joint lesions. A significant lack of strength could result in loss of function and contribute to impairment. The scores assigned by the Medical Research Council (MRC) were as follows:

0 = no contraction; 1 = flicker or signs of contraction; 2 = action motion without gravity; 3 = active motion against gravitation; 4 = active motion against gravity and resistance; and 5 = normal power [8, 9].

(c) *Walking capacity*—Walking ability was evaluated using an observation method, assessing the ability and the level of autonomy of the walk. The capacity to control balance in an upright position, to use the opposite sides of the body alternately, and to conserve energy during displacement are the requirements essential to the execution and maintenance of an autonomous and effective gait path. Deteriorated walking is often significantly associated with pain, weakness, fatigue, joint dysfunction or a significant lack of ROM in the lower limbs [10, 11].

In particular, the permanent loss of ambulatory independence in MCTO is gradual, and the walking pattern can get progressively worse [12].

(d) *Pain*—*Visual Analogic Scale (VAS NRS)* is used to measure and monitor the severity of musculoskeletal pain, duration, frequency and interference with daily living activities in children with MCTO [13].

### ICF domain: activity

(a) *Child Health Assessment Questionnaire (CHAQ)*—It serves as a measure of activity performance. Health status and physical function were assessed as limitations for children and young adults during daily living activities. The CHAQ score is a common tool to measure disability in children, assessing various motor function skills involved in dressing, arising, eating, walking, hygiene, reach, and grip. The possible score range is 0 to 3, with limited mobility defined as a score ≥ 0.13 [14, 15].

(b) *Physical Activity Index (PAI)*—Physical activity and sport participation were measured using a short questionnaire including intensity, frequency, duration and type/mode*.* PAI in a numerical index evaluates the current exercise program by selecting the score for each category (intensity x duration x frequency = score total = less than 20 to 100 sedentary to a very active lifestyle. Activity Category: Sedentary, Poor, Fair, Very good and High) [16–18].

(c) *Modified Rankin Scale (mRS)*—This scale is used to assess the level of disability and severity based on a generic age change, usually used for neurological diseases, but recently also for Osteosclerotic Skeletal Dysplasia [19, 20] (Table 1).

**Table 1:** Modified Rankin Scale for children and adult

Score: Children	Score: Young Adult
0. No symptoms at all	No symptoms at all
1. No significant disabilities despite symptoms; behaviour appropriate to age and normal further development	No significant disabilities despite symptoms; able or carry out all duties activities
2. Slight disability; unable to carry out all previous activities, but same independence as other age- and sex-matched children (no reduction of levels on the gross motor function scale)	Slight disability; unable to carry out all previous activities, but able to look after own affairs without assistance
3. Moderate disability; requiring same help, but able to walk without assistance; in younger patients adequate motor development despite mild functional impairment (reduction of 1 level on the gross motor function scale)	Moderate disability; requiring same help, but able to walk without assistance
4. Moderately severe disability; an able to walk without assistance (in younger patients reduction of at least 2 levels on the gross motor function scale)	Moderately severe disability; an able to walk without assistance and unable to attend to own bodily needs without assistance
5. Severe disability; bedridden requiring constant nursing care and attention	Severe disability; bedridden incontinent and requiring constant nursing care and attention
6. Dead	Dead

### ICF domain: participation

(a) *Canadian Occupational Performance Measure (COPM—Italian version)* is an evidence-based outcome measure studied to identify a patient’s self-perception of performance and the main issues of independence and participation in everyday life [21].

A special ICF-CY checklist has been developed to determine the child’s Activity and Participation in the agencies of everyday living [22, 23] (Table 2).

**Table 2:**
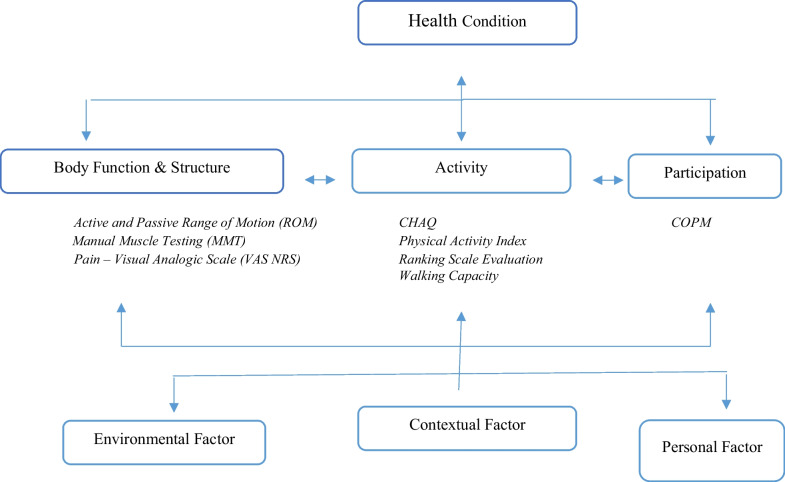
ICF-CY checklist body function-structure and activity-participation

## Results

**Fig. 1:**
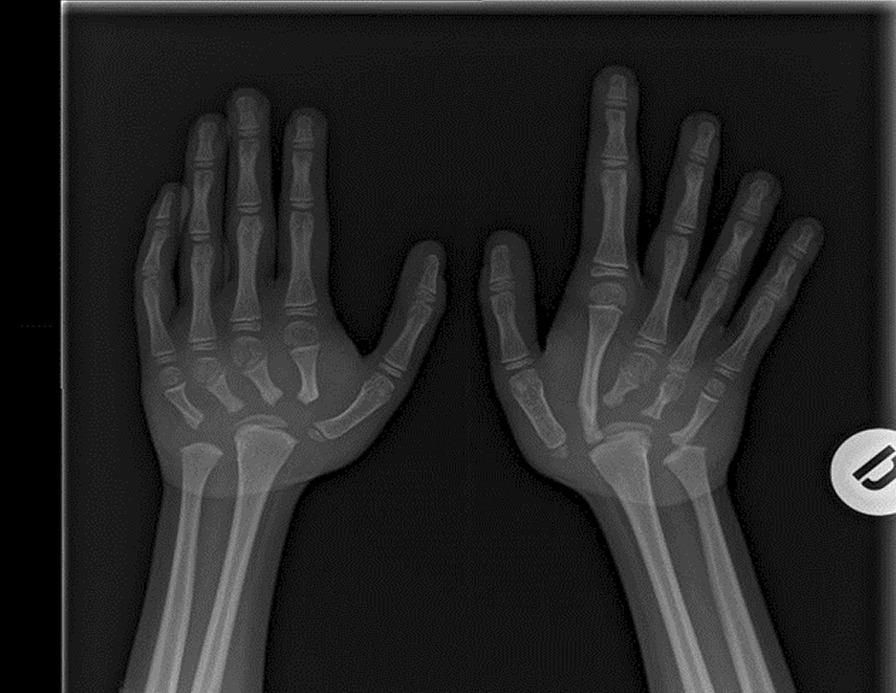
Pt. 6 X-ray at age 9y 11m. Bilateral hand radiograph showing complete resorption of carpal bones and generalized osteopenia

**Fig. 2:**
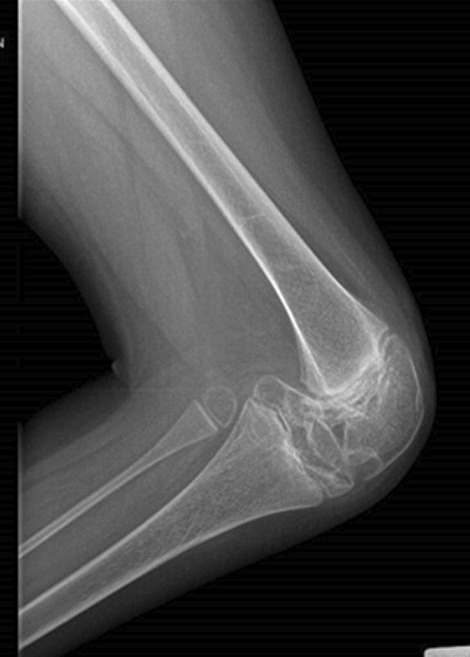
Pt. 2 X-ray at age 11y 6m. Knee radiograph showing important irregularity of the femoral condyles and proximal tibia

**Fig. 3:**
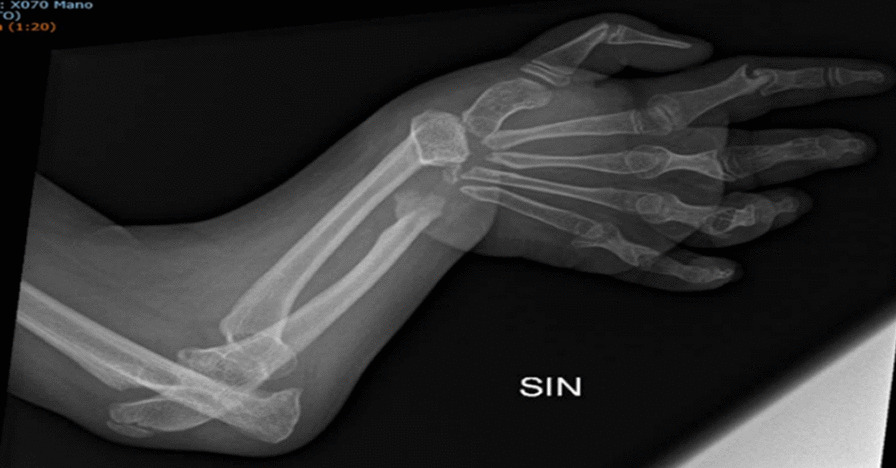
Pt. 2 X-ray at age 14y 5m. Elbow and wrist radiograph showing chronic dislocation with marked dysmorphism of the bony heads, short radius and absence of the carpal bones

**Fig. 4:**
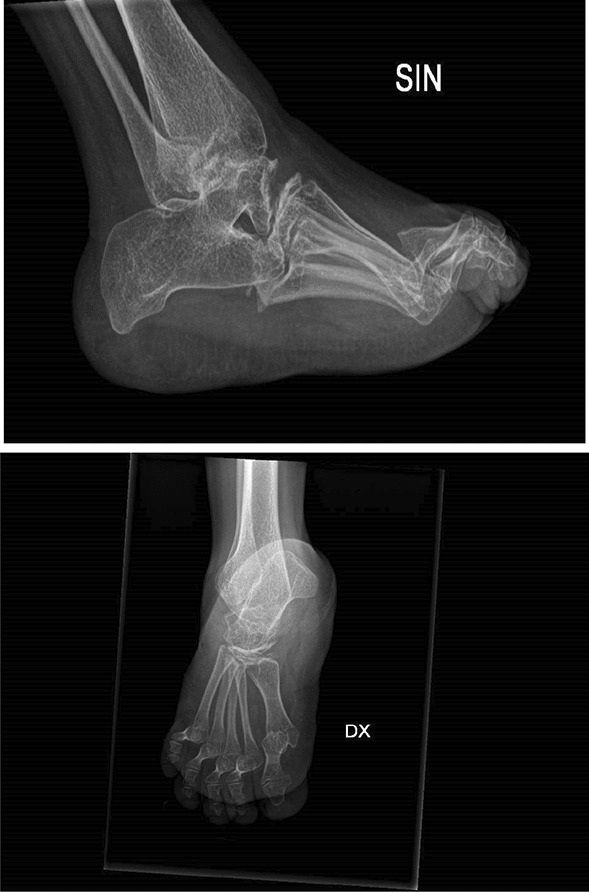
Pt. 2 X-ray at age 16y 6m. Feet radiograph showing complete resorption of tarsal bones and generalized osteopenia

In our case studies, nobody had pain at the time of assessment, once the flare-up phase was over and all patients had aROM-pROM impairment in one or more joints; the wrist was modified in the 6 patients (4 bilateral and 2 unilateral) and there was an ulnar and a radial deviation; 5 patients had a single or bilateral elbow involvement; 3 patients had knee, ankle and foot damage (Table 3) (Figs. 1, 2, 3, 4).

**Table 3:** Clinical history and genetic variant of MAFB in 6 patients with Multicentric Carpo-Tarsal Osteolysis Syndrome

Patient	Age (y,m)	Age at onset (y,m)	Age at diagnosis (y,m)	Autonomous deambulation	MAFB gene variant	First evidence of renal disease (months)	Pharmacological therapies	Others
1	15.8	2.0	6.0	Yes	p.Thr62Ala	60	Denosumab from 2015 to 2018, *ACE-I, B-B	Renal biopsy- focal and segmental mesangial IgM deposit
2	19.11	1.2	1.6	No	p.Pro63Arg	48	Denosumab from 2013 to 2018; ACE-I; NSAID	None
3	12.9	8.8	9.7	Yes	p.Ser70Pro	None	None	None
4	18.6	7.9	10.0	Yes	p.Thr58Ala	132	Methotrexate; Cyclosporine, *ACE-I, B-B	Renal biopsy—interstitial nephropathy with mesangial IgM deposit
5	5.2	1.0	2.7	No	p.Pro71Ala	40	Denosumab from April 2021 to September 2021, *ACE-I	None
6	16.9	1.8	3.0	Yes	p.Thr62Ile	48	Denosumab from 2013 to 2018, ACE-I, Sartans, opioids, joint infiltration of hyaluronic acid	Corneal opacities

Muscle strength, measured using the Manual Muscle Test (MMT), decreased significantly by 4/6 patients in the muscles of the most affected joints in the forearm, wrist, hand and knee. The greater the number of joints, the weaker the strength (Table 4).

**Table 4:** Active and Passive Range of Motion (ROM) and Strength – Upper and Lower Extremity Limitations of 6 patients with Multicentric Carpo-Tarsal Osteolysis Syndrome

Patient	Wrist	Elbow	Knee	Ankle	Foot	Upper Limb/Strenght	Lower Limb/Strenght
1	R/L > 25% a-pROM ulnar deviation R/L	R/L > 50% a-pROM	R/L n	R/L < 10% a-pROM	R/L < 10% a-pROM	MMT 3/5 R/L	MMT 5/5–5/5 R/L
2	R/L > 25%pROM > 50%aROM ulnar deviation R/L	R/L > 75% a-pROM	R/L > 50% a-pROM	R/L > 50% a-pROM	RL > 25% a-pROM	MMT 2/5–3/5 R/L	MMT 4/5–4/5 R/L
3	R > 50%a-pROM L n ulnar deviation R	R/L n	R/L n	R/L n	R/L n	MMT 5/5 R/L	MMT 5/5–5/5 R/L
4	L > 75%pROM R n ulnar deviation L	L < 10% a-pROM R n	R/L n	R/L n	R/L n	MMT 5/5 R/L	MMT 5/5–5/5 R/L
5	R/L > 50%pROM > 75%aROM radial deviation L ulnar deviation R	R/L > 75% a-pROM	R/L > 50% a-pROM	R/L > 50% a-pROM	R/L > 25%a-pROM	MMT 2/5–3/5 R/L	MMT 3/5–3/5 R/L
6	R/L < 25%a-pROM ulnar deviation R/L	R/L > 25%a-pROM	R/L < 25% a-pROM flexed /extended	R/L > 50% a-pROM	R/L < 25% a-pROM	MMT 3/5 R/L	MMT 4/5–4/5 R/L
Total	10/12	8/12	4/12	6/12	6/12		

In the patient group, the CHAQ average was 0.956 (range 0–3); mRS 2.33 (range 0–6): 2 patients had level 4 (moderate-severe inability to walk unaided and inability to take care of one’s own bodily needs without assistance) and all other patients had levels 0–3; and PAI 17.5 (range 1–100), significantly reduced for all patients. The COPM interview was administered to 6 patients. Each patient exhibited a different limitation and priorities in their independence, but, apart from their functional limitation, they showed a relevant agreement in the specific activity of daily life. 5 of them mentioned personal performance problems connected with self-dressing, particularly the management of shirt or pants buttons; 4 of these had problems during the meal such as using a knife to cut meat or peeling an apple or cork from a bottle. 2 patients reported problems with personal hygiene. Only one indicated difficulties and issues related to recreation. Of the 6 patients assessed, one was equipped with an electronic wheelchair, one used a manual wheelchair with an electrical drive and walker, and one used a manual wheelchair only sporadically for long distances. During the interview, it appeared that 5 of them had used or used wrist-hand orthosis. Of these 5, only one kept on using it while the others have gradually abandoned the use. All components used in ICF (Body Functions and Structures, Activities and Participation) are quantified using a generic scale (0–4) (Table 5).

**Table 5:** Function profile of 6 patients with Multicentric Carpo-Tarsal Osteolysis Syndrome

Patient	mRS	PAI	CHAQ	ICF s730 –Body structures of the upper extremity and spine	ICF – s750 Structure of lower extremity	ICF – d450 Walking Capacity	Copm P.	Copm S.	Aids	Orthosis
1	2	16	1,375	s73011.2 s73001.3 s7600.2	s75011.0 s75021.1	d4503.0	4,8	3,4	No	Wrist-hand splints
2	4	1	0,62	s73011.3 s73001.3 s7600.0	s75011.3 s75021.3	d4500.4	6,5	7,5	Electric wheelchair	No
3	1	3	0,25	s73011.1 s73001.0 s7600.1	s75011.0 s75021.0	d4503.0	3,6	2,3	No	Wrist night splint
4	0	48	0,25	s73011.3 s73001.0 s7600.0	s75011.0 s75021.0	d4503.0	10	8	No	Wrist night splint
5	4	1	2,87	s73011.3 s73001.3 s7600.0	s75011.3 S75021.3	d4500.4	3	2,6	Manual wheelchair with power unit	Knee-Foot orthosis Wrist splints
6	3	36	0,625	s73011.3 s73001.2 s7600.0	s75011.2 S75021.3s	d4501.3	6	7	Manual wheelchair for long journeys	Wrist splints

So our data confirm the heterogeneity of the clinical manifestations of the musculoskeletal system of MCTO with important functional limitations and various levels of disability. All patients are affected by at least one body joint. The number of joints involved corresponds to a higher disability and severity evaluation level (CHAQ/mRS). The involvement of the bilateral knee, ankle and foot with a significant deficiency in active and passive ROM is associated with a significant limitation or loss of gait. 4 out of 6 patients can walk autonomously at the right time, while 2 completely lost gait. The only child who reached late walking independence developed the disease around 12 months. The 2 patients who lost their walk were the ones whose lower limb joints were most affected. Hence it can be suggested that greater joint lesions at the lower extremities are correlated with a greater risk of loss of gait. Our patient group has a CHAQ average of 0.956 (range 0–3), mRS 2.33 (0–6) and PAI 17.5 (1–100). Overall, therefore, limitations in daily activities and the resulting impairment are mild to moderate. Physical activity is considerably reduced from what might be expected when correlated with CHAQ and mRS scores.

## Discussion and future perspectives

To define some kind of reliable, reproducible and comparable “function profile” within the same group for each patient, we have tried to measure a variety of global disease outcomes based on the International Classification of Functioning, Disability and Health: Children and Youth (ICF-CY) domains. This profile could be used to define the burden of the disease, its impact on the complexity of the individual and the burden on caregivers. By including more findings in the domains of the ICF model, we would like to have a better functional appearance in MCTO and possibly also in other ultrarare diseases. Perhaps this model could also be used to evaluate the effectiveness of proposed rehabilitation programs, for example, by learning from the outcomes in a domain such as body structure and function, translating into another, such as activity and participation.

Throughout their history of the disease, our patients have all received rehabilitative treatments and in the majority of cases the wrist-hand positioning orthosis was worn. Appraisals show considerable heterogeneity towards rehabilitation. This is due to the presence of a different phenotypical expression of the disease and the difficulty of arriving at a definitive diagnosis. The difference in time between onset and diagnosis does not facilitate the implementation of well-managed rehabilitation [24].

In all cases, every patient at the time of diagnosis requires a functional evaluation that should be repeated over time to adjust the course of rehabilitation. When symptoms of the disease occur at an early stage (less than a year of life), it is crucial to reach the development of motor stages. It supports and assures the acquisition of anti-gravity skills and the achievement of an independent walk.

Evaluations highlight the importance of early rehabilitation management, which may be critical to increasing the independence and participation of the patient with MCTO, limiting the secondary and tertiary damage of the pathology, deconditioning the retractions of the muscle tendons and the loss of movement until immobility. Despite the small numbers, our data may not be significant, but it allows us to focus more on the type and indication of rehabilitation treatment.

Physiotherapy can prevent muscular retraction and atrophy, which can worsen the genetic alteration of ROM and withdrawal. Hydrotherapy can be a complement to many treatment programs to reduce muscular rigidity and allow vertical and active walking assistance, even in severely compromised patients. Both are essential to reach the development of motor milestones when the illness begins in early childhood. Occupational therapy is recommended to determine accommodation measures to support participation in activities relevant to the patient, adjustment of daily living environments and the identification of equipment to support mobility and postural management.

Most of our patients had a postural orthosis during the course of their disease to prevent malformations, especially of the wrist and hand. Ulnar deviation is present in all patients, and significant articular limitations have been established in any case. The use of orthosis does not seem to indicate the effectiveness of preventing malformations [25]. The function of the wrist and hand in these patients is more important than using night splints to ensure wrist and hand alignment such as in juvenile idiopathic arthritis. In the future, in the event of new therapeutic prospects, it may be useful to re-evaluate the description made at this point in their rehabilitation journey.

## Conclusions

We believe it is important to use a comprehensive and longitudinal “function profile” for health and disability. To this end, the ICF-CY approach establishes a common language for describing health and related conditions, enabling the comparison of data collected and a systematic coding scheme. ICF has a comprehensive approach that can be used as a model for other patients with rare diseases or with very specific characteristics (where each case is unique).

MCTO is complex and heterogeneous in its manifestations, although it is simply the involvement of the musculoskeletal apparatus. Lytic lesions, bone loss, generalized osteopenia, posture changes and secondary soft tissue involvement identify functional limitations, loss of independence, and disability with different images that can also be progressive, need to be monitored regularly in time and involve different approaches to rehabilitation. Physiotherapy and occupational therapy are important for the treatment of MCTO symptoms. They are helpful in preventing, reducing or alleviating functional limitations and the resulting disability, trying to maintain the best quality of life for these patients.

We believe it is important to have a complete, periodic and longitudinal functional evaluation of our patients, at the time of diagnosis, after every flare-up and once a year, not only for rehab plans, but also for prescribing devices.

This allows us to monitor the possible local or generalized consequences of MCTO and its impact on autonomy. In our case studies, the functional clinical type, so heterogeneous and so different from one another, but potentially progressive from a disability perspective, implies different approaches to rehabilitation.

### Limits

In the face of the small number of patients with this rare disease, there is a lack of repeated longitudinal monitoring over time for evaluating functional limitations and disability. The age difference at the onset of illness calls for a very different approach to rehabilitation. The difference between symptom onset and diagnosis delays proper rehab management. Hand-selected movement skills have not been assessed because they are complex and therefore require special scales. A focus on patient quality of life does not come with specific questionnaires. Further research is needed to provide an evidence base of best practices in functional clinical assessment and rehabilitation intervention at MCTO.

### Guidelines for the future

First of all, patients need to be physically active. Evaluation should always be equal, repeatable and quantifiable through age and over time to assess the efficacy of current and future treatments. We should try to use the ICF system which gives us the opportunity to measure different domains with a common language in a heterogeneous pathology. Aware that rehabilitation is intended to reduce and contain the secondary damage caused by the disease, it must be understood, once the diagnosis is made, that rehabilitation must aim to improve, support and implement the domains that touch the area of autonomy and participation. Our intervention should always take into consideration the quality of life of patients and caregivers based on real effectiveness.

We would like to see a collaborative effort in the future to design the most comprehensive functional assessment possible for these patients as it relates to their developmental stage, age, disease phenotype and impact on quality of life. These efforts will advance our understanding of the role that available therapies can play, today or in the future, in improving the lives of children and families affected by this and other ultrarare diseases.

## Data Availability

Not applicable.

## Abbreviations

aROM: Active range of motion
CHAQ: Child Health Assessment Questionnaire
COPM: Canadian occupational performance measure
COPM-P: Canadian occupational performance measure-performance
COPM-S: Canadian occupational performance measure-satisfaction
ICF-CY: International classification of functioning, disability and health children and youth
MAFB: Musculoaponeurotic Fibrosarcoma Oncogene Ortholog B
MMT: Manual muscle testing
MRC: Medical Research Council
mRS: Modified Rankin Scale
MCTO: Multicentric Carpo-Tarsal Osteolysis Syndrome
PAI: Physical Activity Index
pROM: Passive range of motion
RANKL: Receptor activator of nuclear factor-kB ligand
ROM: Active and passive range of motion
VAS NRS: Pain-visual analogic scale numeric rating scale
